# Arsenic removal from aqueous solutions by adsorption onto iron oxide/activated carbon magnetic composite

**DOI:** 10.1186/2052-336X-12-58

**Published:** 2014-03-06

**Authors:** Shuhua Yao, Ziru Liu, Zhongliang Shi

**Affiliations:** 1 School of Applied Chemistry, Shenyang University of Chemical Technology, Shenyang, China; 2 GE-HE Wind Energy (Shen Yang) Co., Ltd, Shenyang, China

**Keywords:** Activated carbon, Iron oxide, Arsenic, Adsorption, Magnetic composite

## Abstract

In this work the adsorption features of activated carbon and the magnetic properties of iron oxides were combined in a composite to produce magnetic adsorbent. Batch experiments were conducted to study the adsorption behavior of arsenate onto the synthetic magnetic adsorbent. The effects of initial solution pH, contact time, adsorbent dosage and co-existing anionic component on the adsorption of arsenate were investigated. The results showed that the removal percentage of arsenate could be over 95% in the conditions of adsorbent dosage 5.0 g/L, initial solution pH 3.0-8.0, and contact time 1 h. Under the experimental conditions, phosphate and silicate caused greater decrease in arsenate removal percentage among the anions, and sulfate had almost no effect on the adsorption of arsenate. Kinetics study showed that the overall adsorption rate of arsenate was illustrated by the pseudo-second-order kinetic model. The applicability of the Langmuir and Freundlich models for the arsenate adsorption data was tested. Both the models adequately describe the experimental data. Moreover, the magnetic composite adsorbent could be easily recovered from the medium by an external magnetic field. It can therefore be potentially applied for the treatment of water contaminated by arsenate.

## Introduction

Recognized as a highly toxic element, arsenic (As) is abundant in our environment with both natural and anthropogenic sources [1]. Natural sources include the washout and erosion of arsenic-rich rocks and soils, which probably occur because of long-term geochemical changes. Anthropogenic sources include forestry, agricultural application of various pesticides, herbicides and fertilizers, and industrial effluents from metallurgy, electronics, mining, pharmaceuticals, glass processing, ceramic, dye and pesticides manufacturing, wood preservatives, petroleum refining, and landfill leaching [2,3].

Arsenic occurs in both organic and inorganic forms in natural waters but organic arsenic is of little importance as it goes through biotransformation and detoxifies through methylation. Inorganic arsenic occurs in -3, 0, +3, and +5 oxidation states in aquatic systems. The elemental state -3 and 0 are extremely rare whereas +3 and +5 oxidation states are commonly found in water systems depending on the prevailing redox conditions and pH conditions [4]. Under oxidizing conditions such as those prevailing in surface waters, the predominant species is pentavalent arsenic, which is mainly present with the oxyanionic forms (H_2_AsO_4_^-^, HAsO_4_^2-^) with pK_a_ = 2.19; pK_b_ = 6.94; respectively. On the other hand, under mildly reducing conditions such as in groundwater, As(III) is the thermodynamically stable form, which at pH values of most natural waters is present as non-ionic form of arsenious acid (H_3_AsO_3_, pK_a_ = 9.22) [5]. Inorganic species of arsenic [As(III) and As(V)] represent a potential threat to the environment, human health, and animal health due to their carcinogenic and other effects. Permanent arsenic intake can lead to chronic intoxication, and prolonged arsenic exposure can damage the central nervous system, liver, and skin and results in the appearance of diverse types of cancers, such as hyperkeratosis, lung, skin, and prostate cancers [3,6].

Arsenic contamination has aroused attention due to groundwater levels in many parts of the world at much higher concentrations than the maximum contaminant level (MCL) of 10 μg/L for arsenic in drinking water recommended by the World Health Organization (WHO) [7]. Arsenic pollution has been reported recently in Bangladesh, Taiwan, Argentina, Mexico, Chile, China, Hungary, Thailand, USA, New Zealand, South Africa and India [8-10]. Therefore, an effective arsenic removal technology is thus highly desirable to provide safe drinking water to the affected people. Several methods have evolved over the years on the removal of arsenic present in water and wastewater. These are chemical precipitations, conventional coagulation, reverse osmosis, ion exchange and adsorption. One of which, adsorption method, is simple and cost-effective, thus has been widely used [11-15]. Among various absorbents, adsorption onto activated carbon has proven to be one of the most effective and reliable physicochemical treatment methodologies [16-19]. Due to its high surface area and porous structure it can efficiently adsorb gases and compounds dispersed or dissolved in liquids [20]. The adsorption of several organic contaminants in water, such as pesticides, phenols and chlorophenols, has recently been reported [21-23]. However, the application of activated carbon powders in water treatment system is limited because it is difficult to separate after the treatment process and reuse the tiny particles. The application of magnetic particle technology is one of the choices for field application of the activated carbon adsorbent. Magnetic particles can be used to adsorb contaminants from aqueous or gaseous effluents,and after adsorption, can be separated from the medium by a simple magnetic process.

The application of magnetic particle technology to solve environmental problems has received considerable attention in recent years [24-26]. To our knowledge, the preparation of magnetic composites based on activated carbon and iron oxide and their adsorption properties for arsenic have few been reported so far. In the present work, a series of magnetic composites having high surface area and high adsorption capacity were prepared based on activated carbon and iron oxide. The adsorption of As(V) on the prepared magnetic composites were investigated, the effects of different parameters such as contact time, initial pH, adsorbent dosage and co-existing anionic component on adsorption process were studied, and the optimum adsorption isotherm as well as the rate of adsorption kinetics were found. Compared with other previous reports [27-30], the prime novelties of this work are (1) coating activated carbon onto iron oxide to prepare a magnetic adsorbent; (2) the regeneration of adsorbent was one of key steps to making adsorption technology for practical applications. The separation problem of the prepared adsorbent has been solved, after adsorption, the magnetic composite can be separated from the medium by a simple magnetic process.

## Experimental

### Chemicals

All the chemicals used in the study were of analytical grade. All the solutions in the study were prepared using de-ionized water. All glassware was cleaned by rinsing with hydroxylamine hydrochloride, soaking in 10% HCl, and rinsing with de-ionized water.

As(V) stock solution (1000 mg · L^-1^) was prepared by dissolving dehydrated sodium arsenate(NaAsO_3_) in the de-ionized water. Dissolution of NaAsO_3_ also includes addition of HCl. Further working solutions were freshly prepared from stock solution for each experimental run.

The activated carbon (AC) (AC12 × 40, China Calgon) was used in this study. This kind of AC has moisture content of 1.2%, ash content of 10.3%, iodine values of AC adsorption of 1029 mg/g, the hardness of 96.2%, and the density of 480 g/L. Grain sizes of AC were: less than 1.7 mm in diameter and more than 0.425 mm in diameter. The virgin activated carbon was firstly rinsed with de-ionized water to remove dirties, and then was washed by 0.001 mol · L^-1^ HCl solution to remove all salts precipitated in its pores. Then, the AC was repeatedly washed with de-ionized water to remove all traces of the acid. Subsequently, the washed activated carbon was modified by 10% HNO_3_ for 12 h at the room temperature and repeatedly washed with de-ionized water, finally, the modified activated carbon was oven-dried at 85°C for 24 h to volatilize the organic impurities.

### Adsorbent synthesis

The composite adsorbent used in this study was synthesized using a slightly modified procedure from that reported in the literature [24]. Briefly, the composite adsorbent was prepared from a suspension of the modified activated carbon in a 400 mL solution of FeCl_3_ (7.8 g, 28 mmol) and FeSO_4_ (3.9 g, 14 mmol) at 70°C. NaOH solution (100 mL, 5 mol/L) was added dropwise to precipitate the iron oxides. Later, the obtained material was washed with de-ionized water until rinsing water became neutral, then the adsorbent was dried in an oven at 100°C for 8 h and finally stored in polystyrene bottles for further usage.

### Characterization

The BET specific surface area and pore volumes of adsorbent before and after loading iron oxide were obtained by the cumulative adsorption of nitrogen at 77 K using a Micromeritics 2000 instrument (ASAP 2000, Micromeritics, USA). The point of zero charge (pHpzc) of iron oxide was obtained by interpolating the data to zero EM [31]. The morphologies of iron oxide/activated carbon composite and activated carbon were examined by a scanning electron microscope (SEM, Holland Philips, JSM-5800). X-ray diffraction pattern was taken from a Cu target X-ray diffractometer (Rigaku D/max-r B).

### Batch adsorption experiments

A batch technique was used to investigate As(V) adsorption. Batch experiments included: the kinetic studies, adsorption isotherms and some operating parameters.

The adsorption capacities of activated carbon and iron oxide/activated carbon composite were determined by batch adsorption isotherms at room temperature (20 ± 1°C) in aqueous solution. In several glass vials, 100 mL of solution containing various As(V) concentrations (50, 100, 150, 200, 250 mg/L) were contacted with 5.0 g/L of adsorbent. The vials were placed in a water bath at 20°C and shaken at 150 r/min for approximately 24 h to ensure equilibrium was reached, and the pH was adjusted by adding 0.1 mol/L NaOH or HNO_3_ until it remained constant (±0.10). After filtration through a 0.22 μm membrane filter, the As(V) concentration of the filtered solutions was analyzed with an atomic fluorescence spectrometer (AFS) (PS Analytical Ltd., Kent, UK).

The adsorption kinetic study was performed for As(V) in solution at pH 6.0 and room temperature (20 ± 1°C). Several glass vials were used to hold 50 mL As(V) solution of known initial concentration (2, 5, and 10 mg/L) and 5.0 g/L of composite adsorbent, and shaken at 150 r/min for a duration ranging from 0 to 240 min. At certain period of time, each vial was removed from the shaker, and the solution was then filtered through 0.22 micron filter paper. The filtrates were analyzed for residual As(V) concentration with an atomic fluorescence spectrometer (AFS) coupled with a hydride generator. Arsenic concentration was determined by treating the solution with a reducing agent containing 5% thiourea and 5% ascorbic acid prior to hydride generation and AFS measurement, using a solution containing 1.5% KBH_4_ and 0.3% NaOH as reducing solution and 1% HCl as carrier solution.

To determine the effects of different parameters on As(V) adsorption, experiments were performed at various initial pH, ranging between 2 and 11. Initial concentration of 10 mg/L of As(V) and composite adsorbent dosage 5.0 g/L were employed. The effects of adsorbent dosage and contact time were conducted.

## Results and discussion

### Characterization of adsorbents

**Figure 1:**
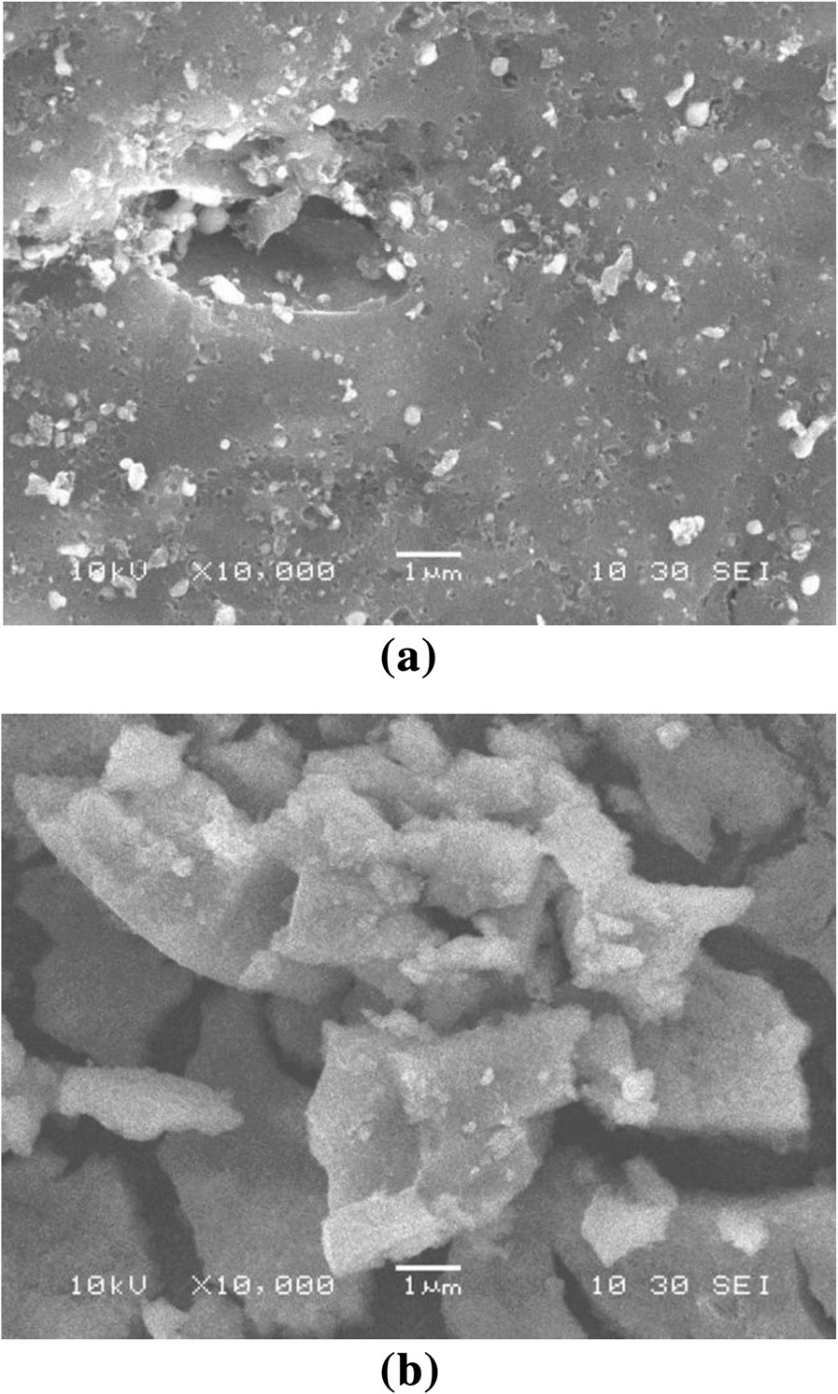
SEM micrographs of activated carbon (a) and iron oxide/activated carbon composite (b).

The microstructure changes of pure iron oxide, pure activated carbon and iron oxide/activated carbon composite adsorbent were listed in Table 1. As shown in Table 1, the deposited iron oxide contributes to a decrease in the N_2_–BET surface area, total pore volume and average pore diameter. As iron oxide has a relatively small surface area and microporous volume (62.8 m^2^/g and 0.009 cm^3^/g, respectively) its presence in the composites should cause a decrease in the surface area and microporous volume compared to pure activated carbon. The point of zero charge (pHpzc) of prepared iron oxide was found to be 7.9.Figure 1 shows the SEM micrographs of activated carbon and composite adsorbent. It could be found from Figure 1 that there are a few macropores in activated carbon, and small aggregates are observed from the general view of the composite, which appear brighter, supported on the darker surface of the activated carbon.

**Table 1:** Microstructure of pure iron oxide, pure activated carbon and iron oxide/activated carbon composite

**Sample**	***S***_**BET **_**(m**^**2**^**/g)**	**Average pore diameter (nm)**	**Total pore volume (cm**^**3**^**/g)**
iron oxide	62.8	0.926	0.009
Activated carbon	1022.6	1.859	0.861
Composite adsorbent	678.3	1.688	0.632

**Figure 2:**
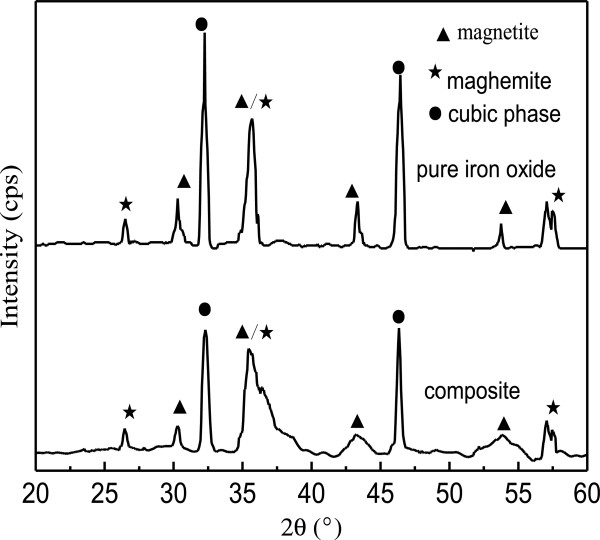
Powder XRD for pure iron oxide and iron oxide/activated carbon composite.

To obtain information on the crystal structure of the prepared composite adsorbent, X-ray diffraction patterns were measured. The XRD patterns of pure iron oxide and composite adsorbent were shown in Figure 2. XRD analyses of pure iron oxide suggest the presence of a cubic iron oxide phase, which may be related to the presence of maghemite (γ-Fe_2_O_3_) and magnetite (Fe_3_O_4_). So the prepared iron oxides are magnetic. For the composite the iron oxide maintained cubic spinel structure. This illuminated that the magnetic properties of iron oxide were basically invariable, which makes the composite adsorbent can be separated more easily by an applied magnetic filter. It could also be seen from Figure 2 that the peaks of cubic iron oxide phase in the composite appear broader, suggesting a smaller crystallite size.

### Effect of initial solution pH

**Figure 3:**
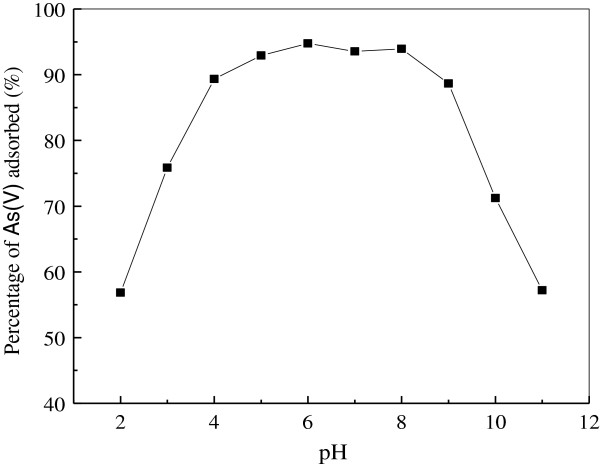
**Effect of solution pH on the adsorption of As(V).** (Experiment conditions employed: initial As(V) concentration 10 mg/L, adsorbent dosage 5.0 g/L, adsorption time 1 h, agitation speed 150 r/min).

The solution pH is an important factor for all water and wastewater treatment processes. Therefore, experiments were performed in order to investigate the effect of initial pH of solution to be treated regarding As(V). Figure 3 shows the percentage of As(V) removed as a function of pH value at pH = 2.0 ~ 11.0.

It is evident that the percentage of As(V) removal strongly depended on the media pH. Furthermore, it can be noticed that the maximum adsorption capacities of composite adsorbent for As(V) occurred at pH 3.0–8.0. Nevertheless, the highest removal efficiency has taken place at pH 6.0 (95.27%) which was chosen as an optimum pH condition for further experiments. The As(V) above the pH value of 3.0 is present in anionic forms and therefore, it can be effectively removed by the iron hydroxides, which at this pH range are present as cationic monomers (Fe(OH)_2_^+^) [32]. Above pH 8.0 As(V) removal was found to be decreased. This observation could be well correlated with the point of zero charge (PZC) of iron oxides. Pure iron oxides, whether they can be identified as having a particular crystal structure or not, typically have PZCs in the pH range 7.0–9.0 [33]. The point of zero charge (pHpzc) of the prepared iron oxide was found to be 7.9. Over the PZC value, iron oxide is present in the monomeric anionic form [Fe(OH)_4_^-^], hence inappropriate for adsorbing anionic components. So the removal of As(V) was suppressed by Fe(OH)_4_^-^ ions that surrounded the surface of the adsorbent by hindering the approach of As(V) to the adsorption sites present on the surface of adsorbent.

### Effect of adsorbent dosage

**Figure 4:**
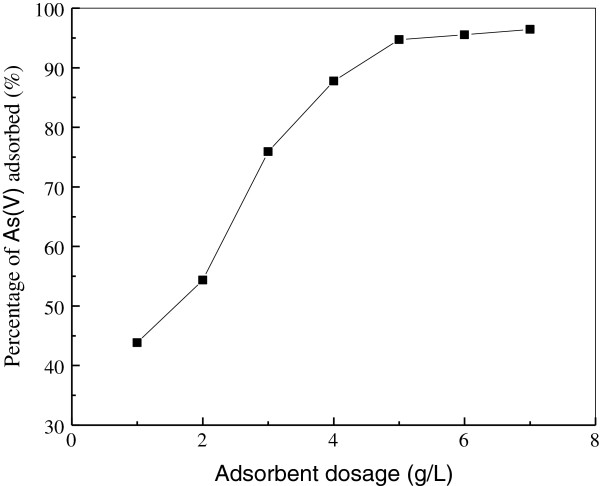
**Effect of adsorbent dosage on the adsorption of As(V).** (Experiment conditions employed: initial As(V) concentration 10 mg/L, solution pH 6.0, adsorption time 1 h, agitation speed 150 r/min).

The effect of adsorbent dosage on percentage adsorption of As(V) was shown in Figure 4. It could be seen from Figure 4 that the removal efficiency of As(V) considerably increased with the increase of adsorbent dosage. The increase in adsorbent dosage from 1.0 to 5.0 g/L resulted in an increase from 25.8 to 89.7% in adsorption of As(V). This may be due to the greater availability of the exchangeable sites or surface area at the higher concentrations of the adsorbent. On the other hand, the increase in the efficiency of removal may be attributed to the fact that with an increase in the adsorbent dosage, more adsorbent surface or more adsorption spots were available for the solute to be adsorbed [3,15,34]. A further increase in adsorbent dosage (>5.0 g/L) did not cause significant improvement in As(V) adsorption. This may be due to the adsorption of almost all As(V) to the adsorbent and the establishment of equilibrium between the As(V) adsorbed to the adsorbent and those remaining unadsorbed in the solution. The results of this study are in accordance with obtained findings by other researchers [2,3,15]. Thus 5.0 g/L of iron oxide/activated carbon composite adsorbent was chosen for next study.

### Effect of contact time

**Figure 5:**
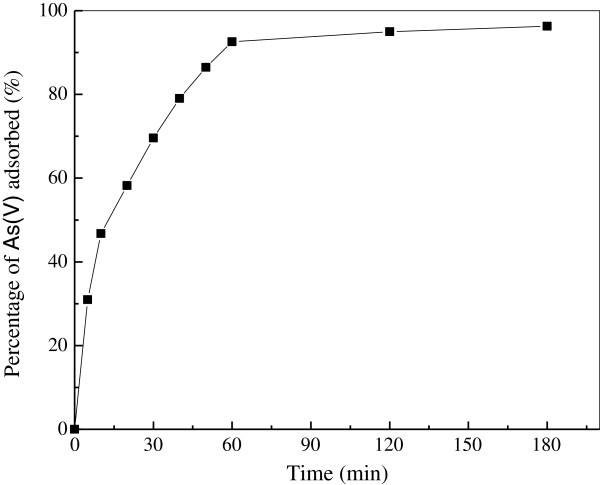
**Effect of contact time on the adsorption of As(V).** (Experiment conditions employed: initial As(V) concentration 10 mg/L, adsorbent dosage 5.0 g/L, solution pH 6.0, agitation speed 150 r/min).

Contact time is one of the effective factors in batch adsorption process. The effect of contact time on As(V) adsorption efficiency was shown in Figure 5. As it is shown, the removal efficiency of As(V) onto the composite adsorbent significantly increase during the initial adsorption stage (0–40 min) and then continue to increase at a relatively slow speed with contact time until a state of equilibrium is attained after 60 min. There was no significant change in As(V) removal rates after 1 h up to 3 h. Based on these results, 1 h was taken as the time in adsorption experiments. Generally the removal rate of sorbate is rapid initially, but it gradually decreases with time until it reaches equilibrium. This phenomenon can be attributed to the fact that a large number of vacant surface sites are available for adsorption at the initial stage, and after a lapse of time, the remaining vacant surface sites are difficult to be occupied due to repulsive forces between the solute molecules on the solid and bulk phases. Similar findings were reported by other researchers [3,35].

### Effect of co-existing anionic component

**Figure 6:**
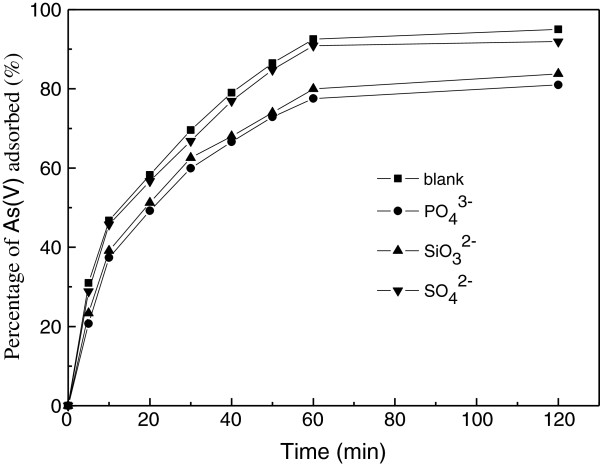
**Effect of co-existing anionic component on the adsorption of As(V).** (Experiment conditions employed: initial As(V) concentration 10 mg/L, adsorbent dosage 5.0 g/L, solution pH 6.0, agitation speed 150 r/min).

In groundwater sources several anionic components might exist, which could compete with arsenic for the available adsorption sites. Among the major co-existing anionic components, sulfate (SO_4_^2-^), phosphate (PO_4_^3-^) and silicate (SiO_3_^2-^) are usually present in groundwater streams possibly inhibiting arsenic removal. In order to investigate the effect of co-existing ions on As(V) removal, arsenic solutions were spiked with SO_4_^2-^, PO_4_^3-^ and SiO_3_^2-^, respectively and the removal of arsenic was determined. At fixed pH of 6.0, the effects of different anions (Figure 6) showed that phosphate or silicate caused the greatest percentage decrease in As(V) removal among the anions. Under the experimental conditions, phosphate resulted in a bigger decrease in As(V) removal than silicate. The effect of sulfate was minimal under the experimental conditions.

It is well known that silicate and phosphate strongly adsorb to metal oxide surfaces via inner-sphere complexation similar to the interaction mode of arsenate with metal oxides. The significant reduction in As(V) adsorption capacity in the presence of SiO_3_^2-^ and PO_4_^3-^ was due to the competition of the anions with As(V) for metal oxides adsorption sites. As(V), silicate, and phosphate are adsorbed on metal oxides through the formation of surface complexes with the surface hydroxyl groups [36]. Sulfate can be absorbed by metal oxides both specifically and non-specifically via inner- and outer-sphere complexation. In addition, the sulfate binding affinity for metal oxides was much weaker than As(V) [37]. Hence, the removal of As(V) is most significantly affected by silicate and phosphate.

### Kinetic study

**Figure 7:**
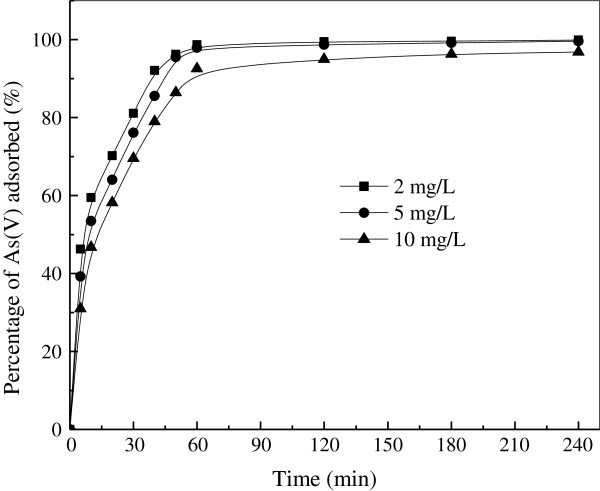
**Adsorption kinetics of As(V) by iron oxide/activated carbon composite.** (Experimental conditions employed: solution pH 6.0, agitation speed 150 r/min, adsorbent dosage 5.0 g/L).

In order to obtain the adsorption kinetic information of As(V) on the iron oxide/activated carbon composite adsorbent, the change of As(V) concentration with adsorption time was recorded for an initial concentration of 2, 5, 10 mg/L. Figure 7 shows the adsorption percentage of As(V) on the composite adsorbent. Obviously, the adsorption is a rapid process, and the equilibrium is reached at 60 min for all three concentrations. For longer periods, adsorption trend seems to remain constant.

In order to investigate the mechanism of As(V) adsorption on the composite adsorbent, the pseudo-second-order rate equation model was applied to the kinetic data. The pseudo-second-order kinetic equation could be derived as [38]:

(1) $$dq_{t}/\mathrm{dt}=k_{2}{\left(q_{e}-q_{t}\right)}^{2}$$

Separating the variables in equation (1) gives

(2) $$-d\left(q_{e}-q_{t}\right)/{\left(q_{e}-q_{t}\right)}^{2}=k_{2}\mathrm{\cdot dt}$$

Integrating both sides for the boundary conditions *t* = 0 to *t* = *t* and *q*_t_ = 0 to *q*_t_ = *q*_t_ gives the integrated rate law for a pseudo- second-order reaction,

(3) $$1/\left(q_{e}-q_{t}\right)=1/q_{e}+k_{2}\mathrm{\cdot t}$$

Equation (3) can be rearranged to obtain:

(4) $$t/q_{t}=1/\left(k_{2}\cdot {q_{e}}^{2}\right)+t/q_{e}$$

The kinetic constant, *k*_2_, can be determined by plotting of *t/q*_t_ against *t*.

The kinetic experimental data of As(V) on the composite adsorbent was simulated by pseudo-second-order rate equation (4). The results were listed in Table 2.

**Table 2:** Kinetic parameters for As(V) adsorption by iron oxide/activated carbon composite

***c***_**0 **_**(mg/L)**	***q***_**e **_**(mg/g)**	***k***_**2**_** (L/(mg.min))**	**R**^**2**^
2	0.4123	0.4035	0.9996
5	1.0389	0.1163	0.9994
10	2.0716	0.0418	0.9995

Remarkably, the kinetic data could be described well by the pseudo-second-order kinetic equation which was based on the assumption that the rate limiting step may be chemical sorption or chemisorptions involving valency forces through sharing or exchange of electron between adsorbent and adsorbate [39]. It could also be seen that the values of the pseudo-second-order rate constant decreased with increasing the initial As (V) concentrations.

### Adsorption isotherms

**Figure 8:**
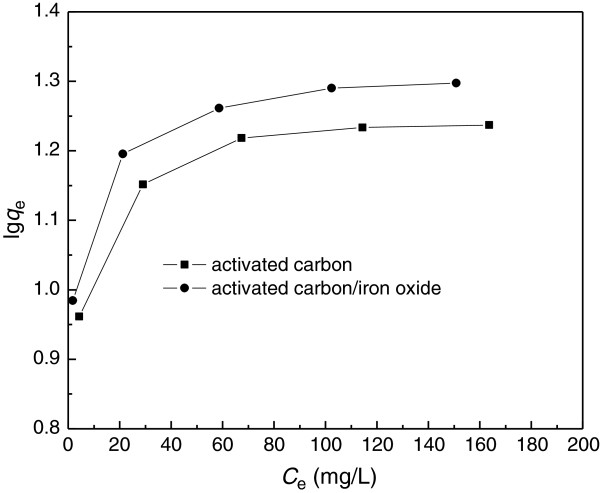
**Adsorption isotherms of As(V) by iron oxide/activated carbon composite. **(Experimental conditions employed: adsorbent dosage 5.0 g/L, solution pH 6.0, adsorption time 24 h, agitation speed 150 r/min).

The adsorption isotherm indicates how the adsorption molecules distribute between the liquid phase and the solid phase when the adsorption process reaches an equilibrium state. Langmuir and Freundlich isotherm equations are the most widely used models to describe the experimental data of adsorption isotherms. As(V) adsorption isotherms obtained for activated carbon and iron oxide/activated carbon composite adsorbent were shown in Figure 8. These isotherms represent the adsorption behavior of As(V) on the different adsorbents as a function of increasing aqueous As(V) concentration for a contact time of 24 h. All the isotherms show that the adsorption capacity increases with increasing equilibrium concentration of As(V), but the increasing slope of iron oxide/activated carbon composite adsorbent is higher than that of activated carbon.The results of As(V) adsorption on all adsorbents (Figure 8) were analyzed by using the Langmuir model to evaluate parameters associated to the adsorption behavior. The linear form of Langmuir equation at a given temperature is represented by:

(5) $$q_{e}=q_{m}\cdot b\cdot c_{e}/\left(1+b\cdot c_{e}\right)$$

where *c*_e_ is the aqueous phase ion equilibrium concentration (mg/L), *q*_e_ is the amount of As(V) sorbet onto 1 g of the considered adsorbent (mg/g), b is the adsorption constant (L/mg) related to the energy of adsorption and *q*_m_ is the maximum adsorption capacity (mg/g).

Equation (5) can be rearranged to obtain:

(6) $$c_{e}/q_{e}=1/\left(b\cdot q_{m}\right)+c_{e}/q_{m}$$

Experimental isotherm data acquired were correlated with the linear form of Langmuir model. The isotherm parameters related to the model were listed in Table 3. It could be seen that both *q*_m_ and b remain the higher for As(V) adsorption onto iron oxide/activated carbon composite. This implies iron oxide/activated carbon composite has a higher adsorption of As(V) than pure activated carbon. High value of b was reflected in the steep initial slope of an adsorption isotherm, indicating desirable high affinity. Therefore, iron oxide/activated carbon performed well in As(V) adsorption.The Freundlich isotherm model was also used to analyze the result of As(V) adsorption on different adsorbents (Figure 8). The Freundlich model can be expressed by the following equation:

**Table 3:** The parameters of Langmuir and Freudlich equation

**Adsorbent**	**Langmuir equation**			**Freundlich equation**		
	***q***_**m **_**(mg/g)**	**B (L/mg)**	**R**^**2**^	**1/n**	**K**_**f**_	**R**^**2**^
Activated carbon	17.86	0.1816	0.9998	0.1808	7.3161	0.9806
Iron oxide/activated carbon	20.24	0.2502	0.9996	0.1642	9.1806	0.9831

(7) $$q_{e}=k_{f}\cdot {c_{e}}^{1/n}$$

where *k*_f_ and n are constants related to the adsorption capacity and affinity, respectively. The equation is conveniently used in the linear form by taking the logarithm of both sides as:

(8) $$\mathrm{lg}q_{e}=\mathrm{lg}k_{f}+\left(1/n\right)\mathrm{lg}c_{e}$$

Experimental isotherm data acquired on different adsorbents were fit with the linear form of Freundlich model and the isotherm parameters related to the model were listed in Table 3. The data showed that the *k*_f_ constant is higher for iron oxide/activated carbon than that for activated carbon, 1/n value for iron oxide/activated carbon composite is smaller than that for pure activated carbon. These imply more favorable adsorption of As(V) on iron oxide/activated carbon composite.

## Conclusion

A magnetic composite adsorbent was successfully prepared with activated carbon and iron oxide as raw materials for the removal of As(V) from solution. The performances of the composite adsorbent were compared to those of pure activated carbon, the composite adsorbent showed fast adsorption kinetics as well as high adsorption capacities. The adsorption properties of the composite adsorbent for As(V) depend on contact time, initial solution pH, adsorbent dosage and co-existing anionic component. The adsorption kinetic data of As(V) can be illustrated very well by the pseudo-second-order rate equation. The equilibrium data obtained from this study was well presented by Langmuir and Freundlich models.

## Competing interests

The authors declare that they have no competing interests.

## Authors’ contributions

YSH was the main investigator, collected the data, performed the statistical analysis, and drafted the manuscript. LZR carried out detailed adsorption and kinetic studies and their interpretation. SZL had done the quantitative analysis of arsenic, extended help in other laboratory studies related to the manuscript and supervised the study. All authors read and approved the final manuscript.
